# Transcriptomic analysis associated with reversal of cisplatin sensitivity in drug resistant osteosarcoma cells after a drug holiday

**DOI:** 10.1186/s12885-019-6300-2

**Published:** 2019-11-05

**Authors:** Divya Niveditha, Harshita Sharma, Syamantak Majumder, Sudeshna Mukherjee, Rajdeep Chowdhury, Shibasish Chowdhury

**Affiliations:** Department of Biological Sciences, Birla Institute of Technology and Science (BITS), Pilani Campus, Pilani, Rajasthan India

**Keywords:** Osteosarcoma, RNA-sequencing, Drug-resistance, Drug-holiday

## Abstract

**Background:**

Resistance to chemotherapy is one of the major hurdles in current cancer therapy. With the increasing occurrence of drug resistance, a paradigm shift in treatment strategy is required. Recently “medication vacation” has emerged as a unique, yet uncomplicated strategy in which withdrawal of drug pressure for certain duration allowed tumor cells to regain sensitivity to the drug. However, little is known about the molecular alterations associated with such an outcome.

**Methods:**

In this study, human osteosarcoma (OS) cells resistant to the extensively used drug cisplatin, were withdrawn from drug pressure, and thereafter cytotoxic response of the cells to the drug was evaluated. We further performed next-generation RNA sequencing and compared transcriptome between parental (OS), resistant (OS-R) and the drug withdrawn (OS-DW) cells. Differentially expressed transcripts were identified, and biological association network (BAN), gene ontology (GO) and pathway enrichment analysis of the differentially regulated transcripts were performed to identify key events associated with withdrawal of drug pressure.

**Results:**

Following drug withdrawal, the sensitivity of the cells to the drug was found to be regained. Analysis of the expression profile showed that key genes like, IRAK3, IL6ST, RELA, AKT1, FKBP1A and ADIPOQ went significantly down in OS-DW cells when compared to OS-R. Also, genes involved in Wnt signaling, PI3K-Akt, Notch signaling, and ABC transporters were drastically down-regulated in OS-DW cells compared to OS-R. Although, a very small subset of genes maintained similar expression pattern between OS, OS-R and OS-DW, nonetheless majority of the transcriptomic pattern of OS-DW was distinctively different and unique in comparison to either the drug sensitive OS or drug resistant OS-R cells.

**Conclusion:**

Our data suggests that though drug withdrawal causes reversal of sensitivity, the transcriptomic pattern does not necessarily show significant match with resistant or parental control cells. We strongly believe that exploration of the molecular basis of drug holiday might facilitate additional potential alternative treatment options for aggressive and resistant cancers.

## Background

Drug resistance remains to be one of the major challenge impeding the success of anticancer therapy in achieving prolonged survival. Multiple mechanisms of drug resistance co-exist within a single patient or even a tumour escaping chemotherapy [1, 2]. According to the classic Goldie–Coldman hypothesis of drug resistance, mutations can be spontaneously acquired by a tumour over time leading to accumulation of drug-resistant clones. Further, resistant variants in a heterogeneous tumor can be selected in a Darwinian process, or a subpopulation of the intrinsically resistant cell might cause re-growth of the tumor [3, 4]*.* Despite diverse mode of action, cancer cells learn to thrive on the drug treatment itself, shrugging off drug scare and continue to grow. Hence, in spite of medical advancements, a vast majority of chemotherapies inevitably fail. Identifying alternative routes by which we can overcome resistance is therefore critical. Interestingly, various pre-clinical studies have reported that cancer cell resistance to a drug is not necessarily linked with genetic mutations associated with drug targets; sometimes the resistant cancers may derive their naturally selected “fitness” through regulation of expression of transcripts post-exposure to drugs that were meant to reduce their fitness [5–9]. Therefore, it is proposed that if these cells are given a ‘drug break’ they eventually might revoke their sensitivity to the drug.

Drug re-challenge has been a well acknowledged old concept. Early studies were reported on drug re-challenge in small-cell lung cancers, various leukaemia’s and also following adjuvant treatment in breast cancer [10–12]. Drug re-challenge has also been in vogue not just in cancers, but also in diseases like a polycystic liver disease where the cessation of treatment led to a rebound in effect [13]. In recent times, similar re-challenge-like routines has been used for the treatment of metastatic breast cancer and platinum-based therapy in ovarian cancers [14, 15]. As conventional therapies are still not completely effective in suppression of tumour growth, and recurrence is a common phenomenon, it is therefore imperative to explore the molecular drivers of the re-sensitization process. Till date, molecular changes associated with drug holiday hasn’t been investigated thoroughly in spite of its potential therapeutic implications. Also, if at all there are forces that allow the sensitivity to reverse, what are the possible molecular events that mark it still remains unknown. Further, the understanding of how to design intermittent therapies, what should be the ideal duration of it for sensitization of resistant cancer types, is least understood.

In our initial study, we characterized the progressive variation in transcriptomic expression pattern of osteosarcoma (OS) cells as it progressed towards acquisition of resistance to cisplatin [16]. OS cells were selected for the study as drug resistance still remains a major obstacle to successful treatment in OS [17–19]. Post RNA sequencing, transcriptomic expression pattern was analyzed and compared between the following samples- untreated tumor cells (OS); non-dividing persisters representing the tolerant cells surviving drug shock (OS-P); extended persisters (OS-EP) denoting the proliferative cells that have revived from drug shock; and the drug resistant cells (OS-R), derived from the OS-EPs, after repeated cycles of exposure to the drug followed by clonal selection of surviving cells. Importantly, the IC_50_ of cisplatin in OS cells was found to be 35 μM, however at similar concentrations only ~ 20% cell death was observed in OS-R cells; the resistant cells thus showed decreased sensitivity to cisplatin at IC_50_ dose compared to parental OS cells [16]. De-regulation of a key network of genes, involved in the regulation of several pathways, was observed in the OS-R cells [16]. As a followup to our initial analysis, in the current study, we wanted to explore whether a drug break reverts the sensitivity in OS-R cells, and if so, what is the molecular basis of such regulation. The resistant cells were cultured under continuous drug pressure; therefore, in this study the OS-R cells were given a drug break and then exposed to cisplatin again to assess sensitivity. Thereafter, next-generation RNA sequencing was performed followed by comparative transcriptomic expression analysis between the drug withdrawn cells (OS-DW), OS and OS-R cells. We provide key insights into molecular events associated with reversal of cisplatin sensitivity in OS cells. This may help in re-orientation of drug treatment strategy against resistant tumor types.

## Methods

### Cell culture and generation of drug resistant cell line

The human osteosarcoma cell line (HOS-CRL-1543) was procured from NCCS, Pune, India, and cultured at 37 °C, 5% CO_2_, in minimal essential medium (HiMedia) supplemented with 10% fetal bovine serum (Invitrogen). The cell line identity was authenticated through STR profiling at Lifecode Technologies Private Limited, New Delhi, India (Project ID: M-1066). The cells were periodically monitored for any contamination. The detailed methodology for the generation of cisplatin resistant OS cells (OS-R) from HOS-CRL-1543 is described in our earlier study [16]. In brief, the parental OS cells were exposed to an acute dose of cisplatin (1 mg/ml) which resulted in survival of a small population of cells; these drug tolerant cells were labeled as “persisters” (OS-P). The persister cells were slow dividing, but over time they revived their population to reach 90% confluency; these cells were termed as the “extended persisters (OS-EP). This cycle of drug exposure followed by revival was repeated to subsequently generate the cisplatin resistant (OS-R) cells [16].

### Establishment of sensitivity in OS-R cells through a drug holiday

The OS-R cells were given a drug break to induce reversal of sensitivity to the drug. These cells were cultured in the absence of the drug cisplatin for seven days. The cell viability assay was thereafter performed to analyze sensitivity to cisplatin.

### Cell viability assay

In vitro analysis of cytotoxicity to cisplatin was evaluated through MTT (3-(4,5-dimethylthiazol-2-yl)-2,5di-phenyltetrazolium bromide) assay following procedures described earlier in Chowdhury et al 2009 [20]. Briefly, cells were cultured overnight and thereafter treated with cisplatin for 24 h. Cells were incubated with MTT followed by DMSO solubilization of formazan crystals formed by the live cells. Readings were taken at 495 nm with a differential filter of 630 nm.

### RNA isolation and quantitative PCR

Total RNA from the cell extracts was obtained through TRIzol reagent (Invitrogen). Complementary DNA (cDNA) synthesis was carried out using GeneSure First Strand cDNA Synthesis kit (Genetix) using oligodT. cDNA for specific genes was amplified and detected using SYBR Green (Bio-Rad) in real time PCR System (Bio-Rad). Livak method was used to quantitate the relative RNA expression level [21].

### mRNA sequencing and analysis

RNA sequencing was conducted at Bionivid Technology Pvt. Ltd., Bangalore using Illumina HiSEQ2500 platform. The raw data was deposited to NCBI’s Gene Expression Omnibus (GEO) with GEO Series accession number GSE86053 [22]. The detailed methodology followed for sequencing and analysis is described in Niveditha et al 2019 [18]. Briefly, cDNA library was prepared and deep sequencing was performed to generate reads which were thereafter aligned to the human genome using TopHat (v2.0.11). Cufflinks was used to obtain expression statistics of each transcript and CuffDiff was used to collect differential gene expression data. Fold change (FC) of 1.5 and above, and a *p*-value cutoff of ≤0.05 was considered for differentially expressed transcripts, whereas, transcripts with log 2-fold change greater than or less than 10 were considered as significantly up or down-regulated respectively. Clustering of transcripts showing differential expression was performed on DAVID server; Cytoscape V 2.8 was applied to visualize the network, and finally, functional annotations were carried out based on Gene Ontology (GO). The differentially expressed transcripts were segregated into three functional domains- receptor-mediated signaling, intracellular signaling, and regulation of intra-cellular processes based on their biological functions, as per GO database. The transcripts showing association with all the three functional domains were considered as “key genes” [16].

## Results

### Resistant OS-R cells regain sensitivity to cisplatin after drug withdrawal

We obtained OS-R cells as described in our previously published study [16]. The OS-R cells were cultured in drug withdrawn media following procedure as described in materials and methods. The sensitivity of the cells (now labelled as OS-DW) to cisplatin, 24 h post treatment was thereafter evaluated. The IC_50_ of OS cells to cisplatin was earlier reported to be ~ 35 μM, at a similar dose, only around 20% cell death was observed in OS-R cells; the IC_50_ of OS-R cells was observed to be ~ 80 μM [16, 23]. However, post culture of OS-R cells in drug withdrawn media the OS-DW cells regained sensitivity to cisplatin. The IC_50_ of cisplatin in OS-DW cells was found to be around 37 μM which was very close to the IC_50_ of cisplatin observed in parental untreated OS cells. We were therefore interested to understand whether the transcriptome of OS-DW cells revert to a pre-treatment state after withdrawal, or there is a unique third state representing the cells after drug holiday. Transcriptomic analysis was therefore performed in OS, OS-R and OS-DW cells and the transcriptomic pattern was compared to understand alterations associated with re-gained sensitivity.

### Comparative transcriptomic analysis between OS-DW with OS-R cells

**Fig. 1:**
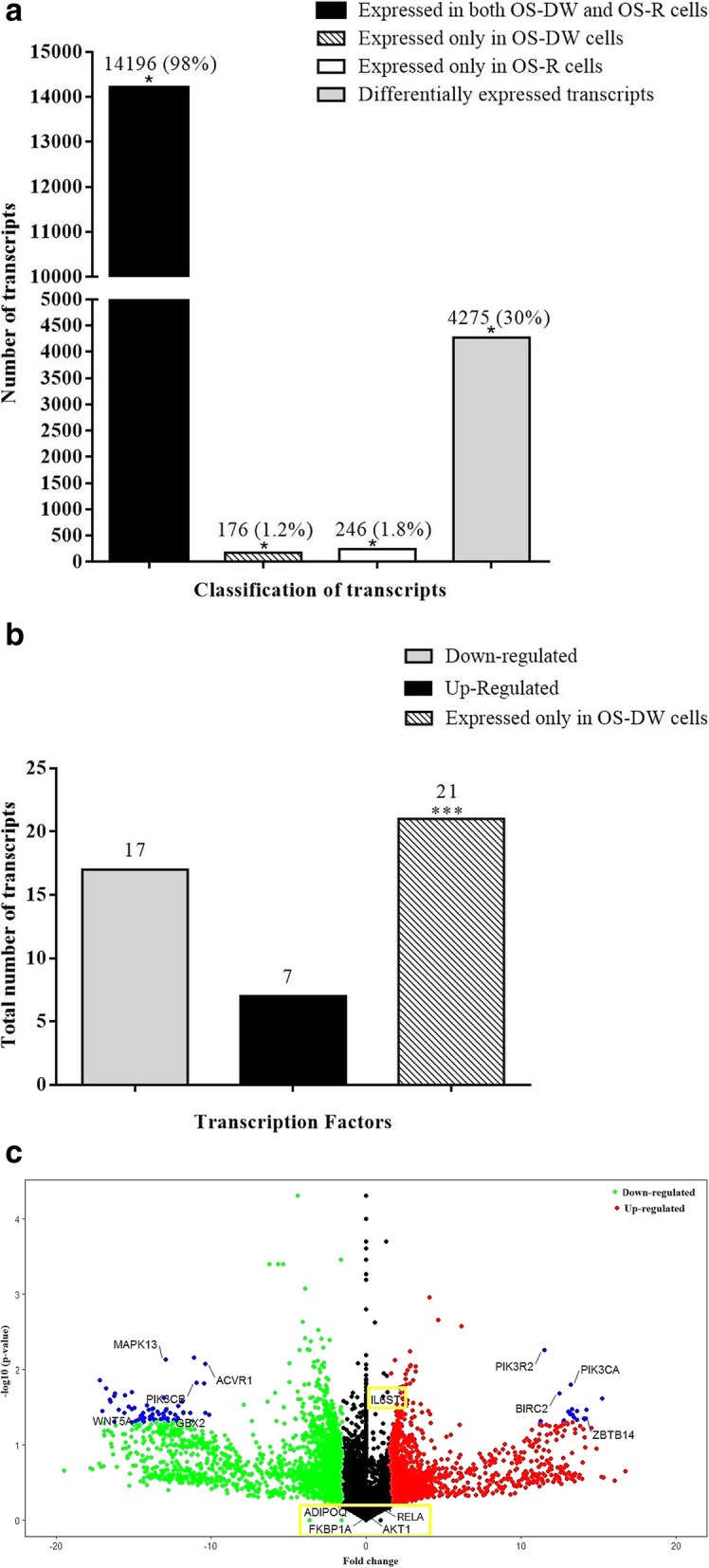
Transcriptomic profiling of OS-DW cells **a)** Bar graph representing distribution of transcripts in drug withdrawn (OS-DW) cells in comparison to resistant (OS-R) cells. The significant differences are indicated by asterisk (*). **b)** The bar graph represents the up and down-regulated transcripts along with treatment-specific transcription factors expressed in response to withdrawal of drug pressure in OS-DW cells. **c)** Volcano plot illustrates the up- and down-regulated genes, in red and green colors respectively, whereas, blue indicates the significantly differentially expressed genes (FC>/< 10) with *p*-values ≤0.05. The genes which are enclosed in the yellow box, represent key genes differentially expressed in OS-R cells, but their expression was seen to be below base-line in OS-DW cells

The distribution of transcripts was analyzed in OS-DW cells and compared with the cisplatin-resistant OS-R cells. A total of 14,196 (~ 98%) transcripts were found to be present in both OS-DW and OS-R cells out of which 4275 (~ 30%) were differentially regulated. Around 176 (~ 1.2%) were expressed only in OS-DW cells and 246 (~ 1.8%) were specific to OS-R (Fig. 1a). The differential expression analysis yielded 2304 (~ 54%) down-regulated and 1971 (~ 46%) up-regulated transcripts. Since, transcriptional de-regulation marked by aberrant expression of transcription factors (TFs) is a key feature of a majority of cancers, we assumed that quantitation of differential expression of TFs, in our samples, might be important to understanding of drug sensitivity in OS cells [24–26]. We observed that out of forty five differentially regulated TFs seventeen were down-regulated, while, seven TFs were up-regulated. Twenty one TFs were expressed only in OS-DW cells compared to OS-R. Some of the transcription factors that were specifically expressed in OS-DW cells were ATF6, CDX2, FOSL1, PRDX3, FZD6, MYNN, TRAF1 which are known to regulate pathways implicated in cancers like, Wnt/Hedgehog/Notch signaling, MAPK signaling, Apoptosis and mTOR signaling (Fig. 1b). Further studies are required to delineate their role in reversal of drug sensitivity. As part of the comparative analysis, the differentially expressed transcripts with log2 FC threshold ≥1.5 and *P* value ≤0.05 are shown in the volcano plot (Fig. 1c). In the volcano plot, we have marked (in blue) the number of genes that showed more than 10 fold differential expression, with a *P* value cut off of ≤0.05. Some of the “key” genes, as per our functional categorization based on annotations in Gene Ontology (i.e., genes regulating intracellular signaling, receptor-mediated signaling and diverse other cellular mechanisms) showed such high differential expression. This included genes like, MAPK13, WNT5A, BIRC2 which are already reported to have a role in tumorigenesis.

### The biological significance of differentially expressed transcripts in OS-DW cells compared to resistant OS-R cells

**Fig. 2:**
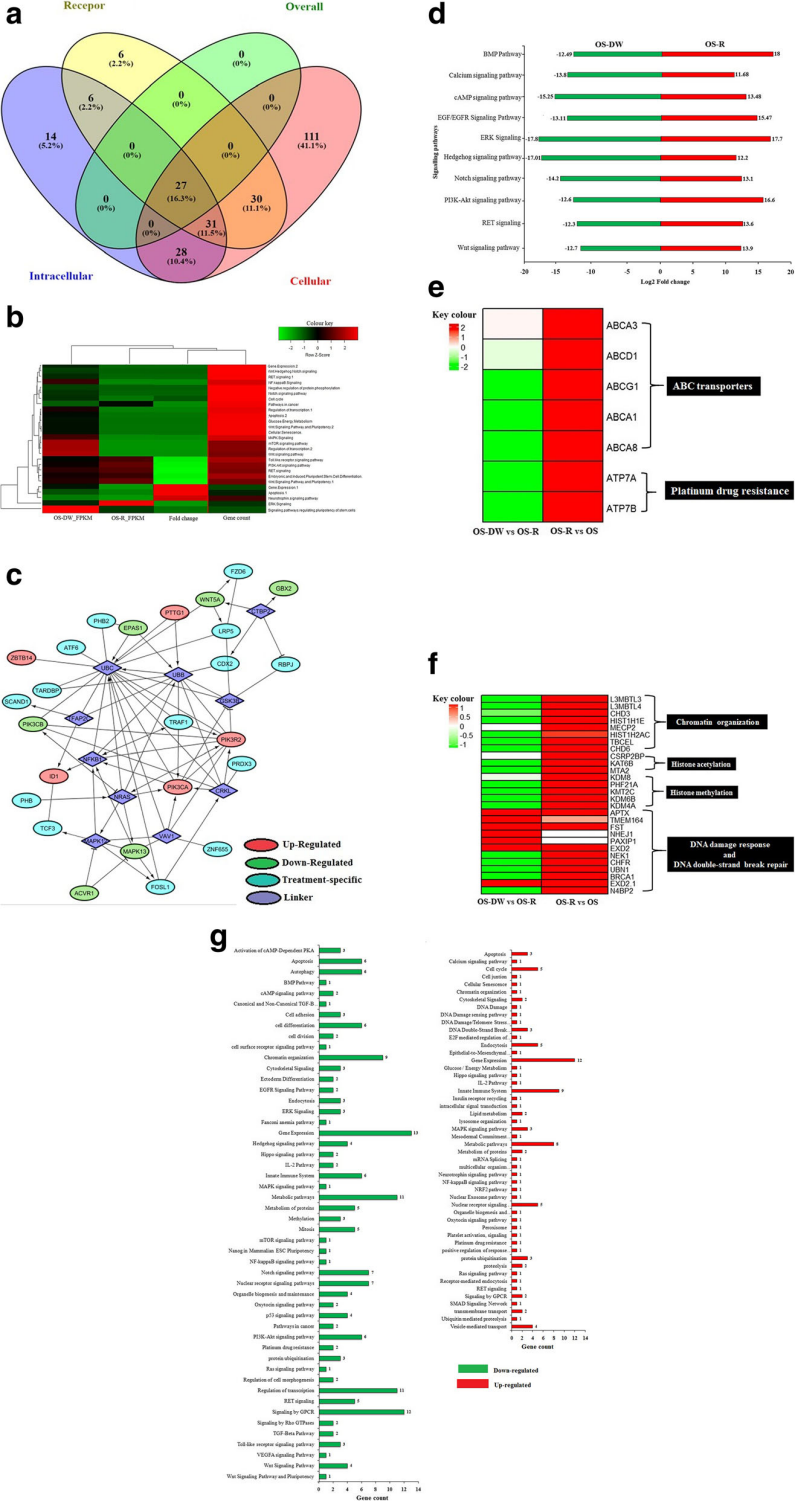
Functional enrichment analysis of differentially expressed transcripts of OS-DW cells **a)** Venn diagram showing number of transcripts involved in regulating different functional domains, categorized as per GO database, in the OS-DW cells, and the number of key genes which are common to all the three domains. **b)** Heatmap representing expression changes of the genes and pathways regulated by the key genes. The RNA-seq expression unit of each sample is taken in FPKM (Fragments Per Kilobase of transcripts per Million mapped reads) **c)** Cytoscape network showing the interaction of up- and down-regulated key genes through genetic linkers of the significant pathways in cancer. **d)** Bar graph representing significantly de-regulated pathways with above FC ≥ 10 in OS-DW cells compared to OS-R cells. Green bar represents down-regulation, whereas, red bars represent up-regulation. **e)** Heatmap showing expression of ABC transporters and platinum drug resistance associated genes in OS-DW cells in comparison to OS-R cells. **f)** Heatmap showing expression of genes associated with chromatin re-organization and DNA repair in OS-DW cells compared to OS-R cells. **g)** Bar graph showing diverse set of pathways that are up- (red) or down (green) regulated in OS-DW cells

We were thereafter interested to identify the transcripts that showed differential expression and map their biological significance. Importantly, key genes that were part of OS-R in comparison to parental OS cells, as identified from our previous report [16], were amongst the ones that were down-regulated in OS-DW suggesting re-orientation of the genetic dependence of the OS-DW cells for their survival upon drug withdrawal. Some of the key genes from OS-R that were down-regulated in OS-DW (represented as box in Fig. 1c) are IL6ST, RELA, AKT1, FKBP1A and ADIPOQ which have well established role in cellular proliferation and/or cancer stemness and associated drug resistance [27–33]. The OS-DW cells, however, had their own subset of key genes; the functional enrichment analysis identified a set of twenty seven key genes in OS-DW compared to OS-R (Fig. 2a). This included genes like, PIK3R2, PIK3CA, BIRC2 and ZBTB14 with implications in cancer [34–38] that were over-expressed with FC above ≥10 in OS-DW in comparison to OS-R; while, expression of WNT5A, PIK3CB, ACVR1, MAPK13 and GBX2 were significantly reduced [39–47] (Fig. 1c). Thereafter, to understand the major pathways that were altered in OS-DW we performed pathway analysis through KEGG mapper taking the key genes as input, which is represented through heat map in Fig. 2b. The pathways involved extended from intra-cellular signalling, stem cell regulation, to control of cellular metabolism and apoptosis, and majority of these pathways have previously been implicated in cancer. Therefore it was imperative for us to dissect deeper into regulation of these pathways. For better visualization of the possible interactions of the up- and down-regulated key genes with other genetic members or linkers of the de-regulated pathways we created an interactive network which is represented in Fig. 2c. Specific linker genes like, GSK-3β, NFĸB were found to form a network with the key genes, that were more predominantly expressed only in OS-DW cells. Interestingly, a further detailed analysis taking differential expressed genes above FC ≥ 10 showed the involvement of genes associated with developmentally active processes like, Hedgehog signalling, Wnt signalling, BMP pathway and Notch signalling to be down-regulated in OS-DW cells suggesting their probable role in imparting resistance. This aspect can be considered in future alongside conventionally used drugs to prevent recurrence or to achieve better sensitization (Fig. 2d). Alongside de-regulation of developmentally active genes, members of key signalling pathways already implicated in multiple cancer types [34, 48, 49] like, EGFR signalling, calcium and cAMP signalling pathway, ERK signalling, PI3K-Akt signalling and RET signalling pathways were also repressed (FC ≥ 10) in OS-DW cells; this might imply altered signal transduction from receptors in OS-DW (Fig. 2d). A major positive impact on resistance to drugs can be exerted by transporter proteins stationed on the cell membrane pumping specific molecules like drugs outside the cell. We observed a significant decrease (FC ≥ 10) in the expression of ABC transporters like, ABCA13 and ABCA8 and platinum drug resistance genes like ATP7A and ATP7B implying their putative role in re-sensitization of the OS-DW cells (Fig. 2e). From the epigenetic perspective, transcripts involved in chromatin organization like lysine demethylases and chromodomain-helicase-DNA-binding protein showed drastic down-regulation in OS-DW cells (Fig. 2f). In contrast to the above subset of genes which mostly showed reduced expression in OS-DW, interestingly, we also observed a set of genes involved in DNA damage response and DNA double-strand break repair (e.g., NHEJ1) to show elevated expression in OS-DW cells in spite of the withdrawal of drug pressure (Fig. 2f). More interestingly, further detailed analysis of DNA repair pathways revelaed up-regulation of majority of genes that are involved in repair pathways such as base excision reapair, mismatch repair, nucleotide excision repair and homologus recombination repair (data not shown). The implication of repair pathways to be up-regulated after drug withdrawal provides hints to their probable role even in absence of drug pressure, and it needs further experimental exploration. Overall, the above analysis clearly demonstrates that the re-gained sensitivity of the tumor cells after drug vacation is resultant of a plethora of changes in expression of genes extending from developmentally active signalling pathways, MAPK pathways, cancer-associated signalling pathways to de-regulation of drug transporters probably facilitated by re-organization at the chromatin level as well. A bar graph showing the diverse set of pathways including a number of genes representing each pathway that were up or down-regulated in OS-DW cells is shown in Fig. 2g. Future identification of key nodes regulating the network of pathways altered upon drug withdrawal might help us identify the novel vulnerability of these tumor cells.

### Comparative transcriptomic analysis between parental OS cells and OS-DW cells

**Fig. 3:**
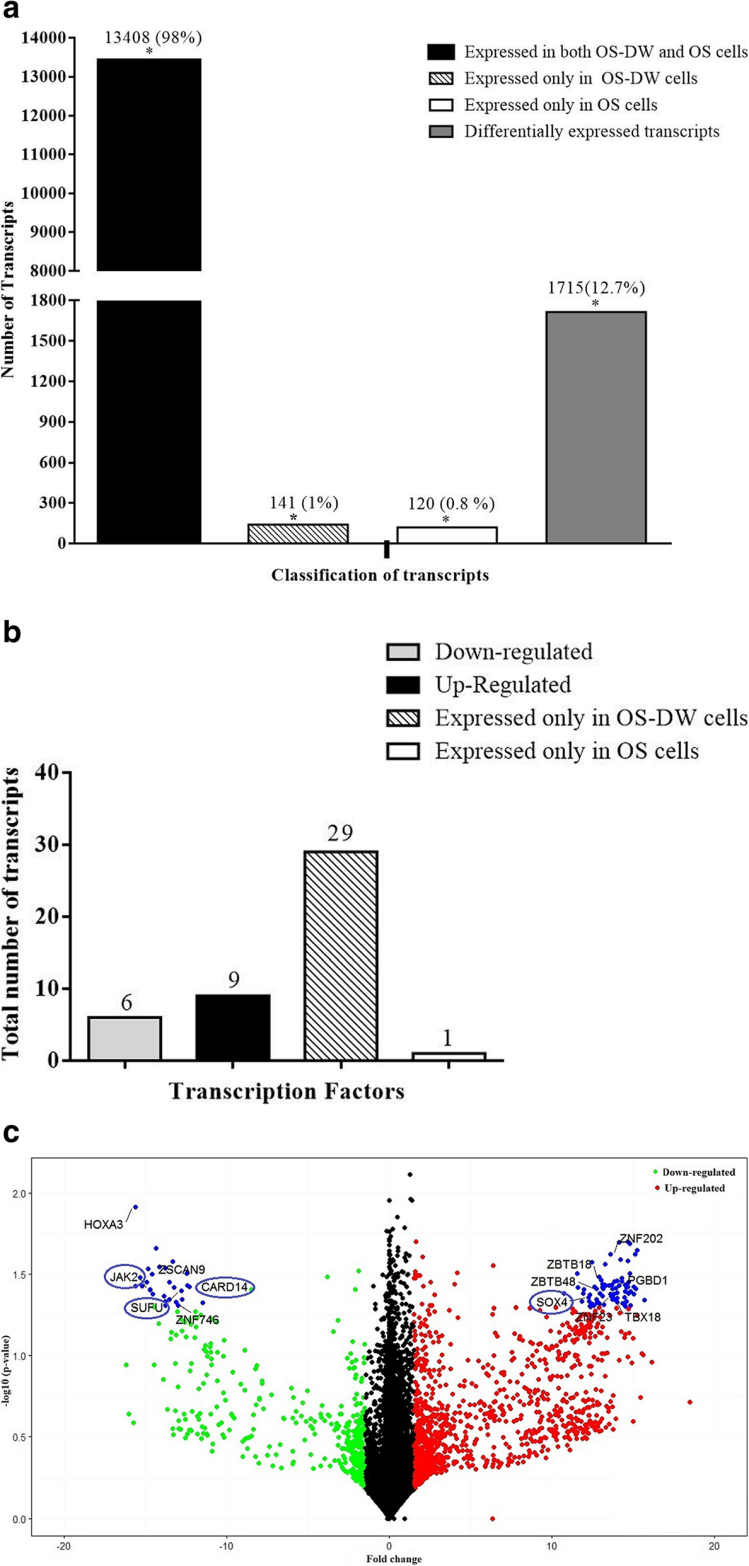
Transcriptomic analysis between OS-DW cells and parental OS cells **a)** Bar graph representing distribution of transcripts in OS-DW cells in comparison to parental cells (OS). The significant differences are indicated by asterisk (*). **b)** The bar graph represents the up- and down-regulated, treatment-specific and control-specific transcription factors. **c)** Volcano plot showing the up- and down-regulated genes, in red and green colors respectively, whereas, blue indicates the significantly differentially expressed genes (FC>/< 10) with p-values ≤0.05. The key genes encircled in blue color are already reported to be involved in regulation of pathways associated with resistance

We thereafter wanted to check whether the drug withdrawn OS-DW cells had any semblance at the transcriptomic level with the parental OS cells; hence, a comparative analysis between parental OS and OS-DW was performed. Our analysis showed a number of transcripts 13,408 (~ 98%) to be expressed in both OS-DW and OS cells (Fig. 3a). Further analysis revealed that 141 (~ 1%) were treatment-specific, expressed only in OS-DW cells and 120 (~ 0.8%) were control-specific, expressed only in untreated OS cells and also a significant number of transcripts 1715 (~ 12.7%) were differentially expressed of which 1291 (~ 75%) were up-regulated and 422 (~ 25%) were down-regulated. We also identified forty five (~ 0.3%) TFs of which fifteen were differentially regulated (six down-regulated, nine up-regulated), twenty nine were treatment-specific and one control-specific (Fig. 3b). The number of treatment-specific transcription factors was significantly high in OS-DW cells. To have a better representation of significantly regulated transcripts, we performed a volcano plot analysis with log2 FC threshold ≥1.5 and *P* value ≤0.05 (Fig. 3c). Around 13 genes were found to be over-expressed with a log2 FC above 10.

### The biological significance of differentially expressed transcripts in OS-DW cells compared to parental OS cells

**Fig. 4:**
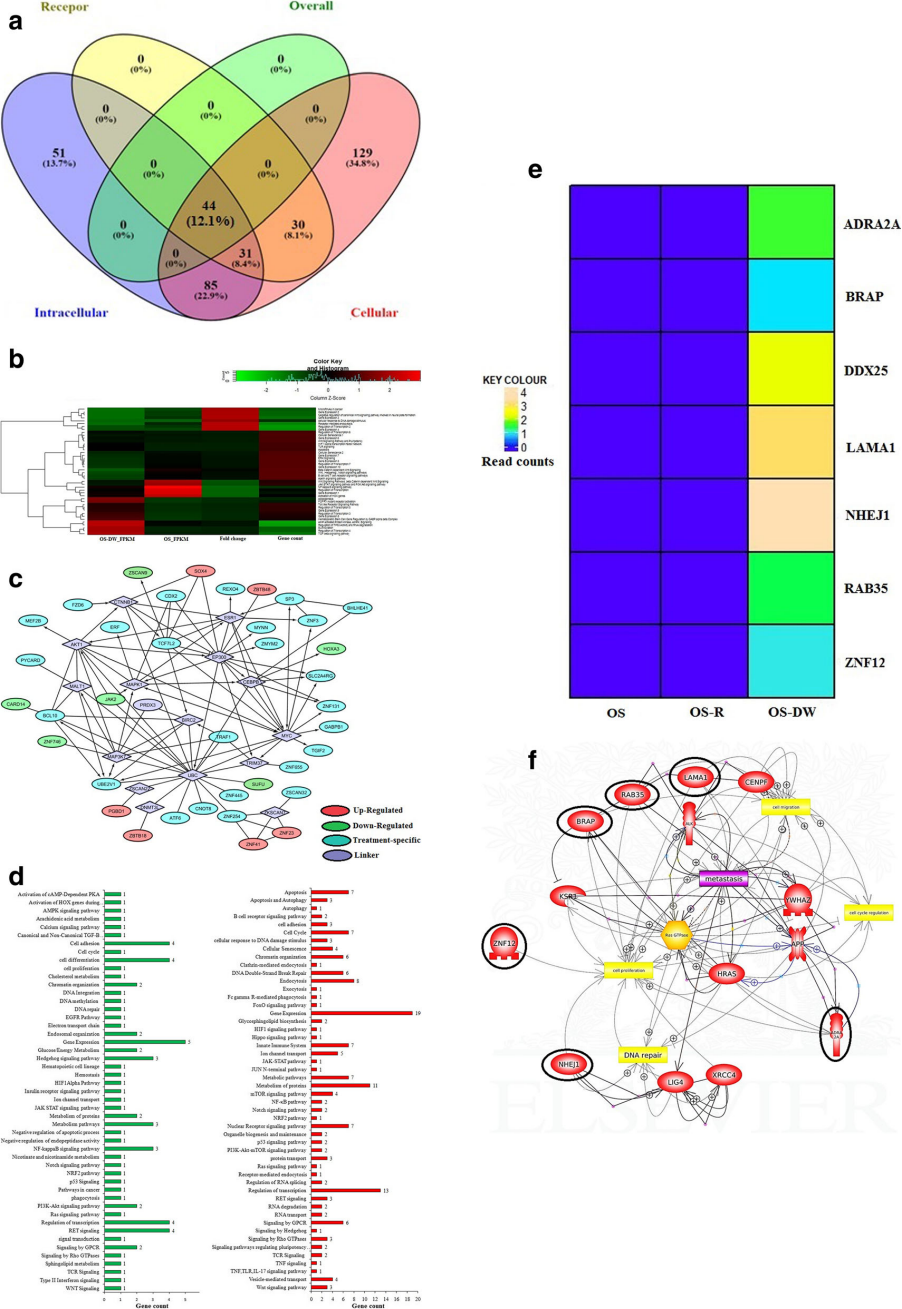
Functional characterization of transcripts of OS-DW cells compared to parental OS cells **a)** Venn diagram showing number of transcripts involved in regulating different functional domains, categorized as per GO database, and key genes that are common to all three domains. **b)** Heatmap representing expression changes of the genes and pathways regulated (also listed in Additional file 1: Table S1a) by the key genes. The RNA-seq expression unit of each sample is taken in FPKM (Fragments Per Kilobase of transcripts per Million mapped reads). **c)** Cytoscape network showing the interaction of up- and down-regulated key genes through genetic linkers of the significant pathways in cancer. All the differentially regulated genes are listed in Additional file 1: Table S1b. **d)** Bar graph showing the diverse set of pathways in OS-DW cells compared to OS cells with FC ≥ 10. The up-regulated are shown in red and down-regulated in green. **e)** Heatmap showing the signature genes of OS-DW cells and their expression in comparison to both OS-R and OS-cells. **f)** The network of the signature genes of OS-DW cells and the pathways they regulate

We functionally categorized the differentially expressed transcripts through GO. This led to the identification of forty four key genes which were common to all the categorized sub-systems in OS-DW compared to OS cells (Fig. 4a). We observed that some of the key genes like, JAK2 (Jak-Stat signalling), CARD14 (NFĸB signalling) and SUFU (Hedgehog signalling) which are reported to be involved in regulating pathways associated with resistance were significantly down-regulated in OS-DW cells compared to OS cells. For deeper insights, we represented the pathways regulated by the key genes through a heat map (Fig. 4b). We have identified the key genes and their pathways in a network generated through Cytoscape (Fig. 4c). The pathways that were up-regulated in OS-DW cells in comparison to OS included the involvement of DNA double-strand break repair pathway, regulation of gene expression and endocytosis which were distinctively different from the other comparison discussed before. A representative bar graph of all the pathways de-regulated in OS-DW in comparison to OS is shown in (Fig. 4d). We further identified a set of genes that were differentially expressed (FC ≥ 10) in OS-DW when compared to both OS-R and OS cells. These genes might reflect the signature of OS-DW cells and is represented through a heat map (Fig. 4e). A deeper analysis of the pathways regulated by these genes showed their involvement in cellular proliferation and metastasis (Fig. 4f). At this stage, we assume these genes might hold the key to the aggressive recurrence of tumor after a drug holiday, however, such speculation is subjected to further investigations.

## Discussion

It is widely accepted that random genetic alterations confer resistance to drugs. However, this fails to explain an increasingly observed phenomenon associated with chemotherapy against cancers- “re-treatment or re-challenge response”. It is proposed that the cells that survive a cytotoxic drug shock, when given a “drug break,” eventually salvage their sensitivity to the drug. The above observation indicates that not necessarily, resistance to drugs is a resultant of stable genetic alterations but may include a reversible state. The potential benefit of treatments with a “drug break” has been addressed in two large phase III clinical trials for docetaxel drug, in androgen-independent prostate cancer patients, where intermittent chemotherapy associated with a drug holiday reduced the level of drug toxicity and improved the quality of life [50–52]. Similar results were also obtained with metastatic renal carcinoma, treatment-resistant melanoma and other cancers, where a drug holiday led to a secondary response. This implies that in patients with a drug break, the resistant tumor cells might shed their resistance property [53, 54]. Therefore, identification of key cellular processes triggered by the withdrawal of drug can be exploited with existing drugs to trigger maximal cell death.

**Fig. 5:**
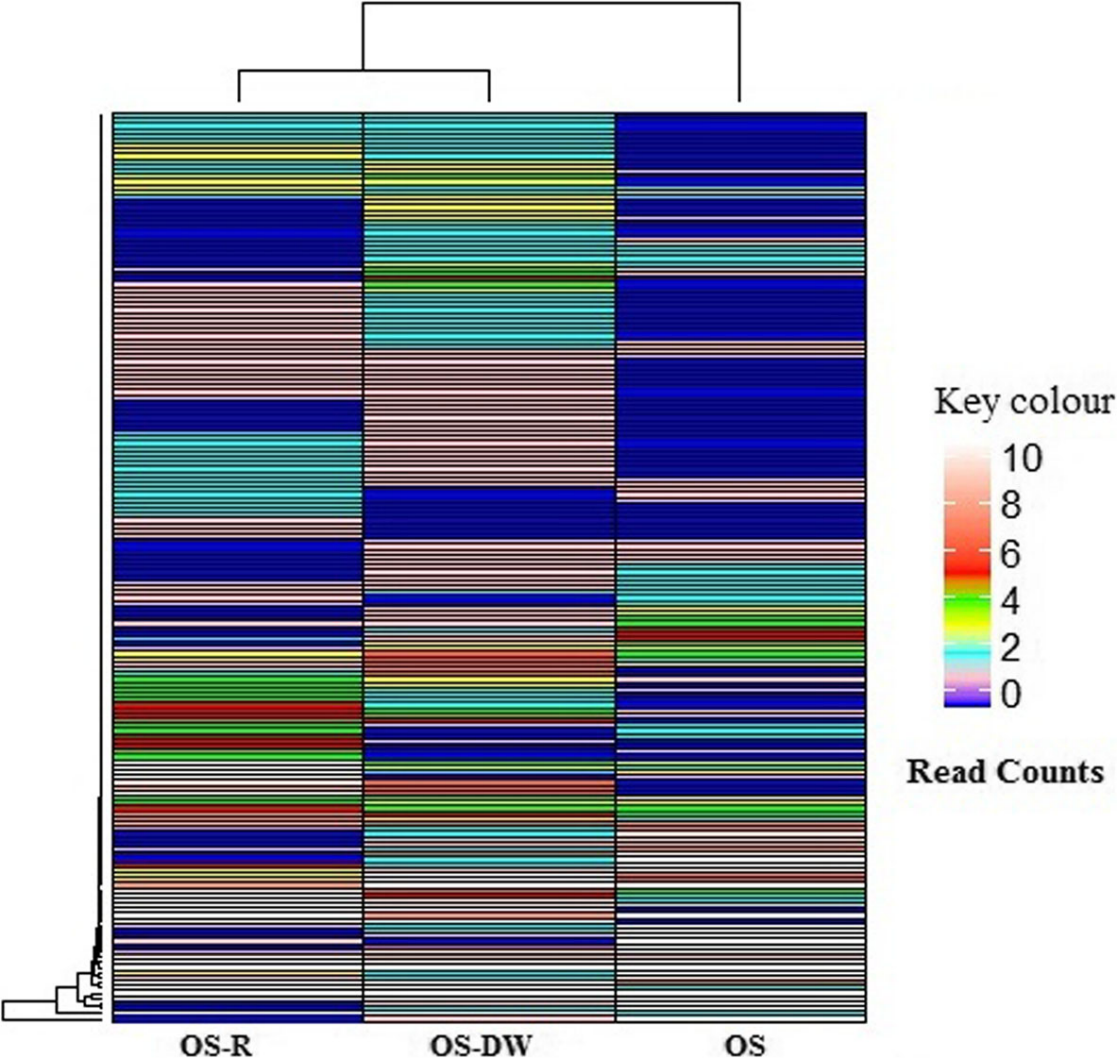
A comparative transcriptomic expression pattern amongst OS-R, OS-DW and OS cells. Heatmap showing read count of transcripts from each group

In this study, we provided a drug holiday to resistant OS cells, and observed a reversal of sensitivity to cisplatin. Thereafter the transcriptomic alterations associated with regained sensitivity was mapped. The key question that we asked was to what extent the resistant cells’ (OS-R) transcriptome differ from the drug withdrawn cells (OS-DW) and what are the key transcriptomic alterations driving reversal of sensitivity. Our results are exciting because they not only portray the importance of drug holiday in the treatment of resistant cancers like, OS, in addition, it also provides molecular information that can be associated with re-sensitization of resistant tumors through a novel therapeutic path. Based on the extensive analysis, we postulate that our drug-withdrawn OS-DW cells has a unique pattern of gene expression and doesn’t have a striking similarity with either the drug sensitive parental (OS) cells or the resistant (OS-R) cells (Fig. 5). After careful analysis of the FPKM value driven color pattern in the heat map, it is clear that majority of the genes in OS-DW is differentially regulated when compared to either OS or OS-R cells. In addition, a very small subset of selected genes was identified to be unaltered between OS-DW and OS cells. Although, we have observed that the expression pattern of a small subset of gene in OS-DW remains to be similar to that of the OS cells, there are significantly high number of genes that are differentially regulated between OS-R and OS-DW and also between OS and OS-DW (Fig. 5). Therefore, it seems that the transcriptome does not necessarily revert to a pre-treatment state after withdrawal, rather there is a unique third state that is attained which is distinctively different from either OS-DW or OS. Indeed, this data identifies that OS-R and OS-DW cells are distantly close to each other, however, they are most likely to have their unique gene expression pattern and thus possible supporting their differential response to cisplatin treatment, even unique than that of the OS cells.

**Fig. 6:**
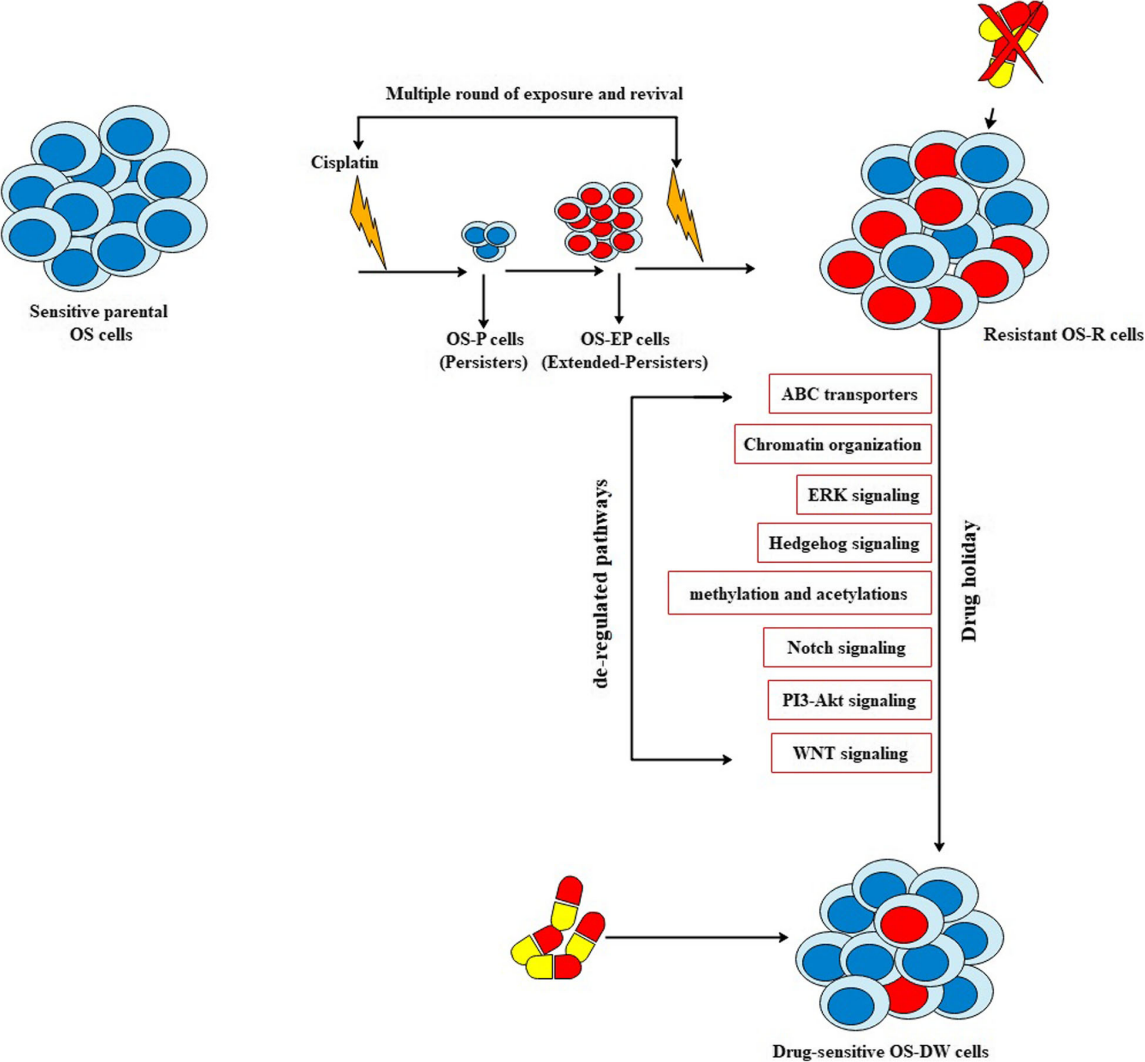
Schematic representation of re-sensitization of resistant cells through a drug holiday

We observed that regained sensitivity in OS-DW cells can not only be attributed to a small set or class of differentially expressed transcripts; rather more than 10-fold differential expression of genes was observed in multiple pathways regulating various critical cellular processes that involved developmentally active signaling, intracellular signaling and chromatin re-organization as well (Fig. 6). We also assume that withdrawal of DNA damaging agent, cisplatin triggers a large build-up of DNA damage response signals which may prove stressful to the OS cells, intensifying tumor cell death after re-treatment. Moreover, a subset of transcripts, that were up-regulated in OS-R [16] like, IRAK3, IL6ST, RELA, AKT1, FKBP1A and ADIPOQ, which are involved in the regulation of critical pathways like, PI3K-Akt signalling, NF-kβsignalling, TGF-β receptor signalling, IL-1 signalling and MAPK pathways were significantly down-regulated in OS-DW cells. This signifies a massive overhaul of transcriptome which might be instrumental in imparting sensitivity to the OS-DW cells again.

Earlier, Sharma et al. in 2010 performed a similar study where drug-sensitive cells were treated with drugs at concentrations exceeding the IC_50_ values [55]. Following multiple rounds of treatments, they detected “drug-tolerant” cells demonstrating reduced drug sensitivity. However, further analyses revealed that the tolerant state was not stable. The tolerant cells-maintained survival by insulin-like growth factor 1 receptor signalling, in an altered chromatin state. In corroboration to above, our study also provides compelling evidence that in OS cells as well, the drug-resistant state is not permanent but is reversible, and the withdrawal of drug pressure is sufficient to induce a transcriptomic make-over effectively re-sensitizing the cells. Hence, this type of “stop and go” strategy provides an appealing treatment option for resistant OS cells. The findings not only have clinical significance, as a drug break may provide respite to the patients from persistent toxic side effects of the drug, but it also has significant economic implications as well, as patients on the intermittent therapy might spend much lesser amount on the anti-cancer drugs, which are overtly expensive, yet achieving clinical remission. However, for some medications, like targeted therapies with drugs for EGFR+ or tumors having multiple metastases before the beginning of first line treatment, continuous therapy might be more favorable than structured treatment interruptions. Therefore, the strategy must depend on the tumor type, stage of the tumor, choice of drugs and also on the patient response. Here in, for the first time we report that resistant osteosarcoma can be re-sensitized by treatment vacations from DNA damaging drugs, like, cisplatin.

## Conclusion

Withdrawal of drug pressure in OS cells facilitates their re-sensitization to the drug- cisplatin**.** The transcriptomic pattern of the drug withdrawn OS-DW cells is unique and do not have striking similarity with either parental OS or resistant OS cells.

## Supplementary information

**Additional file 1: Table S1a.** List of pathways regulated by key genes shown in Fig. 4b. **Table S1b.** List of all the differentially regulated genes mentioned in Fig. 4c.

## Data Availability

All raw data can be accessed from GEO database (https://www.ncbi.nlm.nih.gov/geo/) under the GEO accession number GSE86053.

## Abbreviations

BAN: Biological Association Network
cDNA: Complementary DNA
FC: Fold Change
GEO: Gene Expression Omnibus
GO: Gene Ontology
MTT: 3-(4,5-dimethylthiazol-2-yl)-2,5di-phenyltetrazolium bromide
OS: Osteosarcoma
OS-DW: Osteosarcoma drug withdrawn
OS-EP: Osteosarcoma extended persisters
OS-P: Osteosarcoma persisters
OS-R: Osteosarcoma resistant
TF: Transcription factors
