# Supplementary material for: Transcriptomic analysis associated with reversal of cisplatin sensitivity in drug resistant osteosarcoma cells after a drug holiday
Source: BMC Cancer. 2019 Nov 5;19:1045. doi: 10.1186/s12885-019-6300-2 (PMC6833242; doi:10.1186/s12885-019-6300-2)
Supplement: Supplementary file 1 — Additional file 1: Table S1a. List of pathways regulated by key genes shown in Fig. 4b. Table S1b. List of all the differentially regulated genes mentioned in Fig. 4c. [file 12885_2019_6300_MOESM1_ESM.docx]

**Supplementary Table S1a: List of pathways regulated by key genes shown in Figure 4b**

| Pathways |
| --- |
| MicroRNAs in cancer  Gene Expression 2  Negative regulation of Canonical, Wnt signaling pathway involved in neural plate formation  Gene Expression 3  Cellular response to DNA Damage stimulus  Receptor Mediated Endocytosis  Regulation of Transcription 2  Gene Expression 4  Regulation of Transcription 6  Cellular senescence 1  Gene Expression 6  Wnt signaling pathway/ and pluripotency  HIF.1 alpha transcription factor/ network  TLR signalling  Apoptosis  Cellular senescence 2  Gene Expression 7  ERK signaling  Gene Expression 5  Regulation of Transcription 7  Gene Expression 10  Beta catenin dependent. Wnt signalling  Wnt..Hedgehog..Notch signaling pathway  B cell and T cell receptor signaling pathway  Apelin signaling pathway  Wnt signaling pathway/ Beta catenin dependent Wnt signalling  JAK-STAT signaling pathway/ and PI3K-Akt signaling pathway  NF kappa B signaling pathway  Regulation of Transcription  Gene Expression 1  Activation of HOX gene  Adipogenesis  FGFR1 mutant receptor interaction  Toll like receptor signaling pathway  Regulation of Transcription 5  Gene Expression 9  Regulation of Transcription 3  Gene Expression 8  Hematopoietic stem cell gene regulation by GABP alpha beta complex  AMPK activated protein kinase..AMPK signaling pathway  Regulation of TP53 activity and RNA degradation  PI3K-Akt signaling pathway  SUMOlyation  Regulation of Transcription 4  TGF-Beta signaling pathway |

**Supplementary Table S1b: List of all the differentially regulated genes mentioned in Figure 4c**

| **Up-Regulated** | **Down-Regulated** | **Treatment-specific** | **Linker** |
| --- | --- | --- | --- |
| PGBD1  ZBTB18  ZNF41  ZNF23  ZBTB48  SOX4 | ZSCAN9  SUFU  ZNF46  CARD14  JAK2  HOXA3 | MEF28 FZD6  PYCARD BCL10  UBE2V1 ERF  CDX2 TCF7L2  ATF6 CNOT8  ZNF254 TRAF1  REXO4 MYNN  ZMYM2 SP3  ZNF3 BHLHE41  SLC2A4RG ZNF131  GABPB1 TGIF2  ZNF655 ZSCAN32 | CTNNB1 AKT1  MALT1 MAPK1  PRDX3 MAP37K  ZSCAN22 UBC  ZKSCAN7 MYC  DNMT3L BIRC2  EP300 ESR1  CEBPB |
